# Using the RISK-PCI Score in the Long-Term Prediction of Major Adverse Cardiovascular Events and Mortality after Primary Percutaneous Coronary Intervention

**DOI:** 10.1155/2019/2679791

**Published:** 2019-10-24

**Authors:** Lidija Savic, Igor Mrdovic, Milika Asanin, Sanja Stankovic, Gordana Krljanac, Ratko Lasica

**Affiliations:** ^1^Coronary Care Unit, Clinical Centre of Serbia, Emergency Hospital, Belgrade, Serbia; ^2^Cardiology Clinic, Clinical Centre of Serbia, Emergency Hospital, Belgrade, Serbia; ^3^Center for Medical Biochemistry, Emergency Hospital, Clinical Center of Serbia, Belgrade, Serbia

## Abstract

**Background/Aim:**

The RISK-PCI is a simple score for the prediction of 30-day major adverse cardiovascular events (MACE) and mortality in patients treated with primary PCI (pPCI). The aim of the present study is to evaluate the prognostic performance of the RISK-PCI score in predicting MACE and mortality in the long-term follow-up of STEMI patients treated with pPCI.

**Method:**

The present study enrolled 2,096 STEMI patients treated with pPCI included in the RISK-PCI trial. Patients presenting with cardiogenic shock were excluded. The composite end-point MACE comprising cardiovascular mortality, nonfatal reinfarction and stroke. Patients were followed up at 6 years after enrollment.

**Results:**

One-year and 6-year MACE occurred in 229 (10.9%) and 285 (13.6%) patients, respectively; and 1-year and 6-year mortality occurred in 128 (6.2%) and 151 (7.2%) patients, respectively. The RISK-PCI score was an independent predictor for 1-year MACE (HR 1.24, 95% CI 1, 18–1.31, *p* < 0.001), 6-year MACE (HR 1.22, 95% CI 1.16–1.28, *p* < 0.001), 1-year mortality (HR 1.21, 95% CI 1.13–1.29, *p* < 0.001), and 6-year mortality (HR 1.23, 95% CI 1.15–1.31, *p* < 0.001). The discrimination of the RISK-PCI score to predict 1-year and 6-year MACE and mortality was good: for 1-year MACE c-statistic 0.78, for 6-year MACE c-statistic 0.75, for 1-year mortality c-statistic 0.87, and for 6-year mortality c-statistic 0.83. The nonsignificant Hosmer–Lemeshow goodness-of-fit estimates for 1-year MACE (*p*=0.619), 6-year MACE (*p*=0.319), 1-year mortality (*p*=0.258), and 6-year mortality (*p*=0.540) indicated a good calibration of the model.

**Conclusion:**

The RISK-PCI score demonstrates good characteristics in the assessment of the risk for the occurrence of MACE and mortality during long-term follow-up after pPCI.

## 1. Introduction

ST-elevation myocardial infarction (STEMI) is a complex clinical scenario that requires immediate diagnosis, rapid therapeutic management, and early risk stratification [1]. Primary percutaneous coronary intervention (pPCI) is a reperfusion therapy of choice for the management of patients with STEMI [2–4]. Despite the very low incidence of major adverse cardiovascular events (MACE) after contemporary pPCI, certain patients with STEMI still have an adverse prognosis [1, 2, 5–7]. The identification and quantification of the patient's risk profile is of paramount importance to guide medical management, primarily the duration and intensity of in-hospital care, as well as the proper optimization of therapy during follow-up [1, 8]. Today, it is acknowledged that STEMI patients with high risk of adverse events need more aggressive management than lower-risk patients [8]. Prognosis after STEMI predominantly varies depending on the baseline risk profile; however, echocardiographic and angiographic data are also powerful prognostic variables [1, 2, 4]. Risk scores are mathematical models which include clinical, and in some cases, also laboratory, echocardiographic, and angiographic variables. They are used for estimating the risk of the occurrence of a specific adverse event over a shorter or longer period of time [1]. Several risk scores have been used for the stratification of patients with STEMI, and they can be classified into two groups: risk scores developed in the thrombolytic era and risk scores developed in the pPCI era [1, 9]. The RISK-PCI is a novel, simple score for the prediction of 30-day major adverse cardiovascular events (MACE) and death in STEMI patients treated with pPCI [2, 9]. It has recently been shown that the RISK-PCI score can be used for the prediction of early and late stent thrombosis after pPCI [10]. Although the risk of the occurrence of major adverse cardiovascular events (MACE) and mortality is the highest in the first month after STEMI, for the purpose of devising the best possible treatment plan and providing for secondary prevention, there is a need for assessing the risk of occurrence of these adverse events in the long term, as well. [10–14].

The aim of the present study is to evaluate the prognostic performance of the RISK-PCI score in predicting major adverse cardiovascular events (MACE) and mortality in the long-term follow-up of STEMI patients treated with primary PCI.

## 2. Method

### 2.1. Study Population, Data Collection, and Definitions

The present study enrolled 2,096 patients which were included in the RISK-PCI trial. The design and methods of the RISK-PCI trial have been previously published [2, 15]. In brief, the RISK-PCI is an observational, longitudinal, cohort, single-center trial specifically designed to generate and validate an accurate risk model for predicting MACE after pPCI in patients pretreated with 600 mg clopidogrel. Patients were recruited between February 2006 and December 2009. Informed consent was obtained from all patients. The study protocol conforms to the ethical guidelines of the Helsinki Declaration. It was approved by the local research ethics committee and registered in the Current Controlled Trials Register as ISRCTN83474650 (http://www.controlled-trials.com/ISRCTN83474650). The RISK-PCI study enrolled all consecutive patients, aged >18 years, with clinical and electrocardiographic signs of acute STEMI, within 12 h after the onset of symptoms. The exclusion criteria were refusal to give consent for invasive treatment, active or recent internal bleeding, history of bleeding after nonsteroid anti-inflammatory agents, known bleeding diathesis, intracerebral mass or aneurysm, intolerance or allergy to aspirin or clopidogrel, history of hypersensitivity to iodinated contrast media, cardiogenic shock at admission, noncardiac conditions that could interfere with compliance with the protocol or necessitate interruption of the treatment with thienopyridines, and coexistent conditions associated with a limited life expectancy in the short term. Coronary angiography was performed via the femoral approach. All patients received anticoagulation therapy with unfractionated heparin and dual antiplatelet therapy with aspirin (300 mg) and clopidogrel (600 mg) before the procedure. Flow grades were assessed according to the Thrombolysis in Myocardial Infarction (TIMI) criteria. After pPCI, patients were treated according to current guidelines.

Demographic, baseline clinical, laboratory, angiographic and procedural data were collected and analyzed. Baseline renal function was assessed at admission using the Cockroft–Gault formula. Echocardiographic examination was performed between 48 h and 72 h following pPCI and left ventricular ejection fraction (EF) was assessed according to the biplane Simpson method, in classical two- and four-chamber apical projections.

Patients were followed up at 6 years after enrollment. Follow-up data were obtained by scheduled telephone interviews and outpatient visits. Composite end-point major adverse cardiovascular events (MACE) included cardiovascular death, nonfatal reinfarction, and ischemic stroke. Cardiovascular death included any death due to proximate cardiac cause (myocardial infarction, low-output heart failure, and fatal arrhythmia), sudden death, all procedure-related deaths, and death caused by noncoronary vascular causes, such as cerebrovascular disease. Reinfarction was defined as the presence of (a) an increase in cardiac troponin, above the upper reference limit; (b) recurrent ischemic chest pain, lasting longer than 20 min; and (c) reoccurrence of ST-segment deflection, T-wave inversion, or new pathognomonic Q waves in at least two contiguous leads. Stroke was defined as a new onset of focal or global neurological deficit lasting more than 24 h. Computed tomography was used to classify stroke as ischemic or hemorrhagic. The Emergency Hospital's neurologist was responsible for the diagnosis and treatment of stroke [2].

### 2.2. The RISK-PCI Score

The RISK-PCI score was originally developed and validated to predict 30-day MACE in STEMI patients treated with pPCI. The independent predictors of MACE at 30 days were assigned a risk score based on their regression coefficients. A sum of weighted points for 12 independent predictors was calculated to define the total score for each patient with a range of 0–20. Risk strata with low (0–2.5 points), intermediate (3–4.5 points), high (5–6.5 points), and very high (≥7 points) risk classes were defined to optimize the discrimination ability of the model [2].

### 2.3. Statistical Analysis

Continuous variables were expressed as median values with 25th and 75th quartiles, whereas categorical variables were expressed as frequency and percentage. Analysis for normality of data was performed using the Kolmogorov–Smirnov test. Baseline differences between groups were analyzed using the Mann–Whitney test for continuous variables, and the Pearson *X*^2^ test for categorical variables. The Cox proportional-hazards model was used to assess the value of the RISK-PCI score as a predictor for 1-year and 6-year MACE and mortality. Adjustments were made for variables that were shown to be independent predictors of 1-year and 6-year MACE and mortality in the univariate analysis (age, Killip class >1 at admission, EF, leucocytes count at admission, anterior infarction, and 3-vessel disease). Discrimination of the model (capability to discriminate between true-positive and false-positive outcomes) was measured by c-statistics using the area under the ROC curve (AUC) as an index of model performance. Calibration or difference between predicted and observed events (goodness-of-fit) was assessed using the Hosmer–Lemeshow *X*^2^ estimates. The Kaplan–Meier curves were used to present MACE-free and survival probability during follow-up according to RISK-PCI score classes.

A probability value of less than 0.05 was considered statistically significant. SPSS statistical software, version 19.0, was applied (SPSS Inc, Chicago, IL) (Tables 1 and 2).

## 3. Results

Out of a total of 2,096 patients, 1,529 (72.9%) were men and 567 (27.1%) were women. The median age of all analyzed patients was 59 years (51, 69). The total 6-year follow-up was completed in 2056 (98.2%) patients. One-year and 6-year MACE occurred in 229 (11.1%) and 285 (13.6%) patients, respectively; and 1-year and 6-year mortality occurred in 128 (6.2%) and 151 (7.3%) patients, respectively. Demographic, clinical, laboratory, and angiographic characteristics of analyzed patients according to the occurrence of MACE and mortality at one year and six years are presented in Table 3.

After multivariate adjustment, the RISK-PCI score remained an independent predictor for 1-year MACE (HR 1.24, 95% CI 1.18–1.31, *p* < 0.001), 6-year MACE (HR 1.22, 95% CI 1.16–1.28, *p* < 0.001), 1-year mortality (HR 1.21, 95% CI 1.13–1.29, *p* < 0.001), and 6-year mortality (HR 1.23, 95% CI 1.15–1.31, *p* < 0.001). Independent predictors for mortality and MACE are shown in Table 4.

The discrimination of the RISK-PCI score to predict 1-year and 6-year MACE and mortality was reasonably good. The c-statistics for 1-year MACE prediction was 0.78 (95% CI 0.73–0.79, *p* < 0.001); the c-statistics for 6-year MACE prediction was 0.75 (95% CI 0.68–0.75, *p* < 0.001); the c-statistics for 1-year mortality prediction was 0.87 (95% CI 0.84–0.89, *p* < 0.001); and the c-statistics for 6-year mortality prediction was 0.83 (95% CI 0.78–0.86, *p* < 0.001).

**Figure 1:**
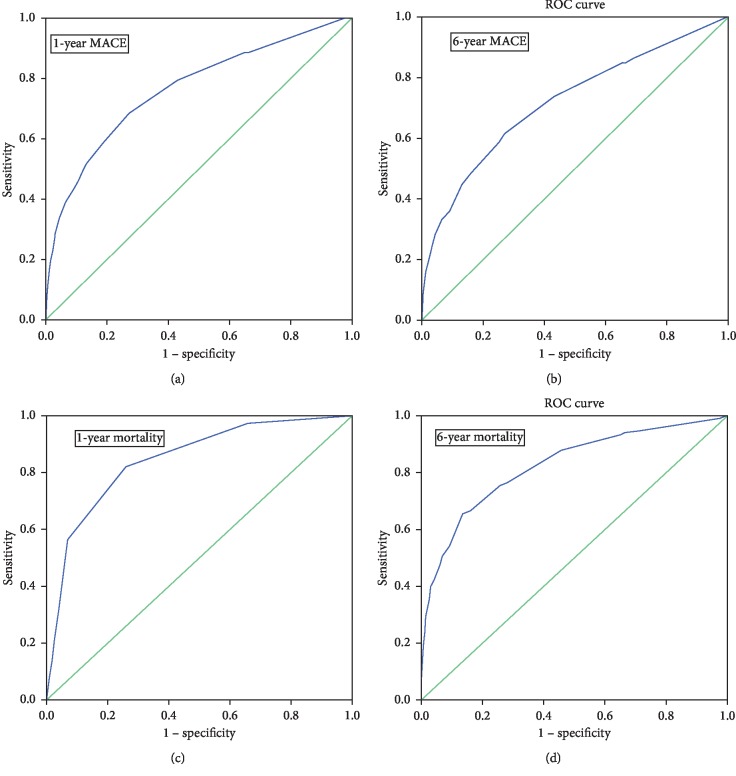
The ROC curves of the RISK-PCI score in predicting 1-year MACE (curve a), 6-year MACE (curve b), 1-year mortality (curve c), and 6-year mortality (curve d). (a) AUC = 0.78. (b) AUC = 0.75. (c) AUC = 0.87. (d) AUC = 0.83.

The discrimination of the RISK-PCI score is shown in Figure 1.

**Figure 2:**
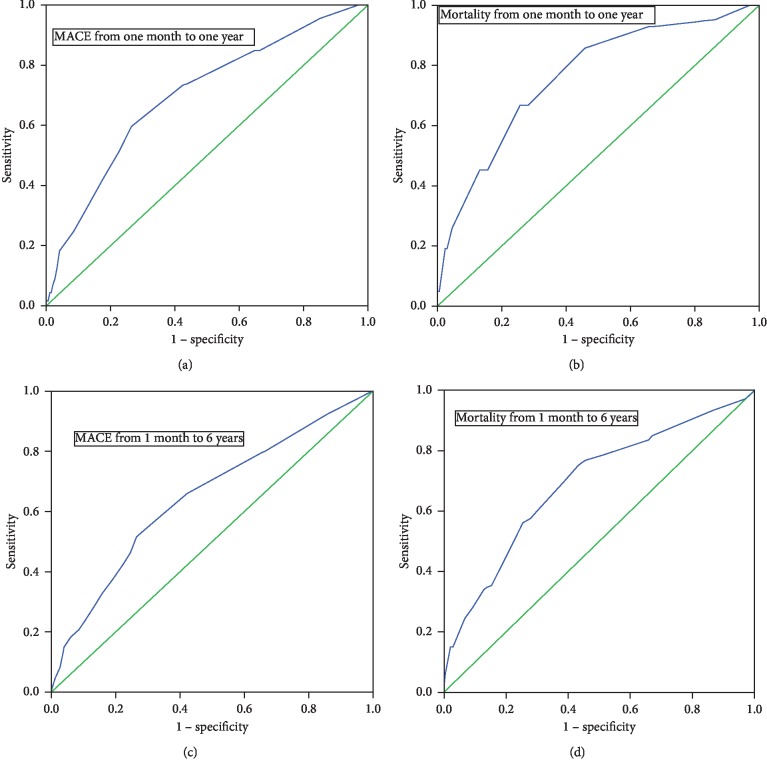
The ROC curves of the RISK-PCI score in predicting MACE and mortality from one months to one year (curves a and b) and from one month to six years (curves c and d). (a) AUC 0.71, 95% CI 0.66–0.75, *p* < 0.001. (b) AUC 0.76, 95% CI 0.68–0.84, *p* < 0.001. (c) AUC 0.65, 95% CI 0.61–0.71, *p* < 0.001. (d) AUC 0.71, 95% CI 0.63–0.76, *p* < 0.001.

The predictive ability of the RISK-PCI score from one month to one year and 6 years is shown in Figure 2.

In addition, the nonsignificant Hosmer–Lemeshow goodness-of-fit estimates for 1-year MACE (*X*^*2*^ = 4.429, *p*=0.619), 6-year MACE (*X*^*2*^ = 7.019, *p*=0.319), 1-year mortality (*X*^*2*^ = 7.373, *p*=0.258), and 6-year mortality (*X*^*2*^ = 5.027, *p*=0.540) indicated a good calibration of the model.

**Figure 3:**
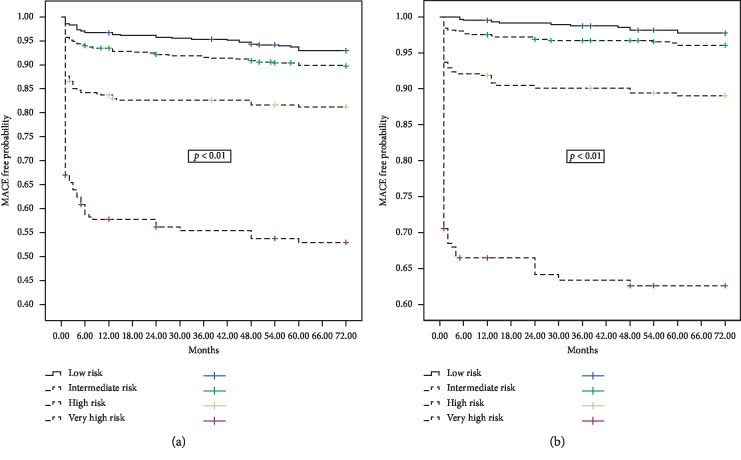
Six-year MACE-free (curve a) and survival probability (curve b) according to RISK-PCI score risk class.

Figure 3 shows Kaplan–Meier curves of MACE-free and mortality probability during follow-up in the 4 risk strata.

## 4. Discussion

The results of the present study have shown the RISK-PCI score to have a satisfactory discrimination ability and predictive value in the assessment of risk of the occurrence of MACE during 1-year and 6-year follow-up of patients with STEMI treated with pPCI. The RISK-PCI score has excellent characteristics in assessing the risk of the occurrence of 1-year and 6-year mortality in these patients. When we analyzed the score characteristics from 1 month up to a year and up to 6 years, we found the sensitivity and specificity of the score to be lower, which we explain to be due to a lesser number of patients with MACE and a lesser number of mortality outcomes after a period of 1 month.

To the best of our knowledge, the RISK-PCI is currently the only risk score which can estimate the probability of the occurrence of MACE and mortality upon STEMI during long-term follow-up of up to 6 years. The discrimination ability of the RISK-PCI score in assessing the risk of MACE occurrence is satisfactory, albeit lower than the discrimination ability of the score in assessing the risk of mortality. Due to heterogeneity in different endpoints, models for composite endpoints have been more difficult to create. In this context, a lower discrimination ability could be expected as compared to mortality models. Also, the discrimination ability of the RISK-PCI score to assess adverse events during 30-day follow-up is better as compared to the discrimination ability of the score to assess the same events during a longer follow-up period [2]. It is usual for the discrimination ability of the risk score to decrease with the length of patient follow-up; however, in most scores, it remains satisfactory up to a certain point of follow-up (which may be a year, 2, 3, etc.). One of the possible explanations for this finding lies in the fact that the largest percentage of adverse events in patients with STEMI occurs in the first few months after discharge from hospital and remains relatively stable thereafter [16]. However, we nevertheless feel that there is a group of high-risk patients who continue to be at a high risk of the occurrence of MACE and/or mortality even after the first month has elapsed.

In a study by Littner et al., the characteristics of the CADILLAC, GRACE, PAMI, TIMI, Dynamic TIMI, and Zwolle scores in assessing risk of mortality and rehospitalization due to acute decompensated heart failure (ADHF) were tested over a follow-up period of up to 3 years, in patients treated with pPCI. Although all the above mentioned scores were initially constructed for risk assessment in short-term follow-up, they have all demonstrated a satisfactory discrimination ability in long-term follow-up; however, it decreased with the increase of the length of patient follow-up, which is in keeping with the results obtained in the present study. In the study by Littnerova et al., the best characteristics for assessing the 3-year mortality risk were demonstrated by the GRACE score and then by the CADILLAC, PAMI, Dynamic TIMI, Zwolle, and TIMI scores, respectively. As to the risk of rehospitalization due to ADHF, the best characteristics were demonstrated by the CADILLAC score [16]. As opposed to our study, in the study by Littner et al., the characteristics of the said scores for assessing the risk of the occurrence of MACE were not tested.

In addition to baseline clinical characteristics, the RISK-PCI score also includes echocardiographic and angiographic characteristics, which makes it different from most of the other risk scores which refer to patients with STEMI [2, 9, 16, 17]. It has been demonstrated that the risk scores which include both clinical and angiographic variables have an improved prognostic accuracy as compared to risk scores which include only clinical or only angiographic variables [4, 6, 12, 13]. In addition to predictors of great prognostic importance, such as age, anterior infarction, bundle branch block, renal dysfunction, ejection fraction, and postprocedural flow TIMI <3, which were present in previous scores, the RISK-PCI score includes variables that were not used in earlier scores, such as previous infarction, complete AV block at admission, glucose intolerance, leukocytosis, postprocedural flow <1, and small vessel size [9, 16]. These variables are well-known predictors of adverse events upon STEMI, both in short-term and long-term follow-up [12, 16]. On the other hand, the RISK-PCI score has been developed without taking into account the variables such as heart failure or heart rate at admission, which may offer incremental prognostic information [7, 9, 12, 16, 18].

The satisfactory characteristics of the RISK-PCI score in the assessment of the risk of the occurrence of adverse events in long-term patient follow-up may be explained by the fact that it had been constructed on the basis of the analysis of data from the RISK-PCI observational study, which included all consecutive patients with STEMI treated with contemporary pPCI. This manner of constructing the risk score is not unusual (e.g., the Zwolle score was constructed in this way). However, the majority of risk scores related to patients treated with pPCI (CADILLAC score, PAMI score, etc.) have been constructed through post hoc analysis of data from randomized pPCI studies. Models derived from clinical registries (or observational studies) enrolling consecutive patients are theoretically more applicable to real-life patients than those developed from patients enrolled in clinical trials, which tend to exclude high-risk patients (very old patients, patients with comorbidities, etc.) [8]. This is why the incidence of mortality and other adverse events in the population of patients enrolled in randomized clinical studies is lower than in the general population of patients with STEMI. Therefore, it is considered that the scores obtained by analyzing data from randomized studies may overestimate the risk of mortality or MACE occurrence, both in short-term and long-term follow-up [1–3, 6].

Also, in order for the risk assessment of adverse events to be proper, especially in long-term follow-up, it is important that the population of patients which the risk score is based on be treated in the same way as the population of patients that the risk score will be applied on. This primarily refers to the application of therapy which has proven to have beneficial effect on the patients' prognosis [1]. In patients with STEMI, this implies that they have been treated with primary pPCI, as well as that they have been taking dual antiplatelet therapy for a sufficient length of time, which is important when assessing the probability of the occurrence of ischemic events in long-term follow-up [2, 10]. In the RISK-PCI study, all of the patients received loading doses of aspirin and clopidogrel, and the average length of dual antiplatelet therapy application was 10 ± 2 months, which makes the RISK-PCI score adequate for the population of patients who are nowadays treated with primary PCI [19]. On the other hand, previously published pPCI scores were derived from trials which did not at all apply dual antiplatelet therapy [20] or did not use clopidogrel before pPCI [21, 22]. When looking at the risk scores obtained in the thrombolytic era, it should first be reiterated that primary PCI achieves a higher percentage of reperfusion success in comparison with thrombolysis, and therefore, beneficially affects the patient's prognosis, i.e., decreases mortality, reduces the risk of the occurrence of new ischemic events, improves the quality of life, etc. Also, STEMI patients treated with pPCI generally have different clinical characteristics in comparison with patients treated with thrombolysis [1, 6, 9]. Therefore, many authors agree that original risk models from the thrombolytic era may not be relevant in most patients managed according to current guidelines [1, 9, 14].

### 4.1. Clinical Implications

Risk assessment for the occurrence of adverse events in long-term follow-up may be useful for the planning of further patient treatment and for secondary prevention [9, 23]. Secondary prevention following STEMI is a very important issue because further ischemic events after the index event are common [24]. The possible clinical significance of evaluating the risk of the occurrence of adverse ischemic events in long-term follow-up, exceeding a year, is the identification of patients with a high risk of the occurrence of ischemic events, who would be candidates for the application of prolonged dual antiplatelet therapy (more than 12 months), taking into consideration, of course, the possible hemorrhagic complications [11].

### 4.2. Study Limitations

Prognostic assessment was derived using a single-center database. The intent was not to compare the efficiency of the score of the present study with previously published scores of PCI patients. In keeping with the widely accepted risk models for primary PCI [1], patients with cardiogenic shock at presentation were excluded from the trial. By definition, these patients fall into the highest risk category and their treatment differs from the overall pPCI population [19]. Also, the protocol of the study stipulated that patients with cardiogenic shock at admission should have separate risk stratification and a different treatment strategy [2, 18]. Patients with cardiogenic shock at admission were also excluded from the studies where the most prominent risk scores for patients treated with pPCI were constructed (e.g., CADILLAC and PAMI) [6]. In the present study, patients were treated with clopidogrel; there were no patients treated with more recently developed antiplatelet drugs (prasugrel and/or ticagrelor); and pPCI was predominantly performed using bare-metal stents. Ticagrelor, prasugrel, and/or the new generation of drug-eluting stents or biodegradable polymers were not available for routine administration to patients at the time of their enrollment into the trial, and this could have influenced the prognosis of the analyzed patients. This study was not designed to compare the characteristics of the RISK-PCI score with that of other risk scores related to patients treated with primary PCI, neither to perform the external validation of the model.

## 5. Conclusion

The RISK-PCI score demonstrates a good discrimination and predictive value in the assessment of the risk for the occurrence of major adverse coronary disease and mortality during long-term follow-up of up to 6 years, in patients with STEMI treated with primary PCI. This simple risk score could be of use to doctors in planning further patient treatment (after hospital discharge), carrying out secondary prevention programs and rehabilitation. Further studies are warranted to externally validate this model and confirm the results from the present study.

## Figures and Tables

**Table 1:** RISK-PCI score.

Risk factor	Points
Age >75 years	1
Prior infarction	1.5
Anterior infarction	1
Complete AV block^*∗*^	2
Acute BBB^*∗*^	3.5
Leukocyte >12.0^10−9^/L^*∗*^	1
Glucose ≥6.6 mmol/L^*∗*^	1

Creatinine clearance^*∗*^	
≥90 ml/min	0
60–89 ml/min	1
<60 ml/min	2

LV ejection fraction <40%	1.5
Reference diameter ≤2.5 mm	1
Preprocedural TIMI flow 0	1
Postprocedural TIMI flow <3	3.5
^*∗*^At admission	

**Table 2:** Risk stratification.

Risk class	Score, points	MACE, observed (%)	MACE, expected (%)
(1) Low	0–2.5	1.9	0.7–3.5
(2) Intermediate	3–4.5	5.9	5.0–8.8
(3) High	5–6.5	13.3	10.7–18.2
(4) Very high	≥7	39.4	22.3–95.0

**Table 3:** Baseline characteristics of analyzed patients according to 1-year and 6-year MACE and mortality.

Variable	1-year MACE			6-year MACE			1-year mortality			6-year mortality		
	No *N* = 1867	Yes *N* = 229	*p* value	No *N* = 1811	Yes *N* = 285	*p* value	No *N* = 1968	Yes *N* = 128	*p* value	No *N* = 1945	Yes *N* = 151	*p* value
Age, med (IQR)	58 (51, 68)	63 (54, 73)	<0.001	59 (51, 68)	63 (54, 73)	<0.001	58 (51, 68)	71 (61, 78)	<0.001	59 (61, 68)	71 (60, 77)	<0.001
Males (%)	1361 (72.9)	154 (67.1)	0.031	1320 (72.9)	193 (67.9)	0.027	1435 (72.9)	80 (62.3)	0.003	1418 (72.9)	96 (63.5)	<0.001
Previous MI (%)	189 (10.1)	39 (17.2)	<0.001	177 (9.8)	49 (17.1)	<0.001	203 (10.3)	24 (18.6)	<0.001	198 (10.2)	26 (17.5)	<0.001
Previous PCI (%)	48 (2.6)	9 (3.8)	0.179	45 (2.5)	11 (3.8)	0.149	49 (2.5)	6 (4.4)	0.143	53 (2.7)	5 (3.6)	0.376
Diabetes (%)	353 (18.9)	63 (27.4)	<0.001	323 (17.8)	77 (27.1)	<0.001	356 (18.1)	43 (33.8)	<0.001	354 (18.5)	48 (31.9)	<0.001
Hypertension (%)	1249 (66.9)	163 (71.2)	0.164	1188 (65.6)	208 (72.9)	0.013	1303 (66.2)	92 (71.6)	0.310	1328 (68.3)	111 (72.9)	<0.001
Hyperlipidemia (%)	1150 (61.6)	116 (50.8)	<0.001	1111 (61.4)	155 (54.4)	0.009	1214 (61.7)	52 (40.9)	<0.001	1241 (63.8)	67 (44.6)	<0.001
Smoking (%)	1041 (55.8)	89 (38.9)	<0.001	1010 (55.8)	120 (42.1)	<0.001	1092 (55.5)	39 (30.6)	<0.001	1120 (57.6)	50 (33.3)	<0.001
Family history (%)	640 (34.3)	15 (6.4)	0.002	625 (34.5)	75 (26.3)	0.001	673 (34.2)	29 (20.2)	<0.001	498 (25.6)	21 (20.3)	<0.001
Pain duration med (IQR)^*∗*^	2.5 (1.5, 4.5)	3.5 (2, 6.5)	<0.001	2.5 (1.5, 4.5)	3.5 (2, 6.5)	<0.001	2.5 (1.5, 4)	3 (2, 5.5)	<0.001	2.5 (1.5, 4.5)	3 (2, 6.5)	<0.001
KillipII and III at admission (%)	183 (9.8)	89 (38.9)	<0.001	176 (9.7)	95 (33.3)	<0.001	193 (9.8)	74 (57.9)	<0.001	196 (10.1)	75 (49.6)	<0.001
Systolic pressure^*∗∗*^ (IQR)	140 (120, 150)	130 (110, 150)	<0.001	140 (120, 150)	130 (110, 150)	<0.001	140 (120, 130)	130 (105, 150)	<0.001	140 (120, 150)	130 (105, 145)	<0.001
Heart rate^*∗∗*^ (IQR)	80 (70, 90)	80 (70, 100)	<0.001	80 (70, 90)	80 (70, 110)	<0.001	77 (68, 90)	90 (75, 110)	<0.001	80 (70, 90)	85 (70, 110)	<0.001
Anterior MI, *n* (%)	721 (38.6)	134 (58.4)	<0.001	697 (38.5)	154 (54.1)	<0.001	773 (39.3)	77 (60.1)	<0.001	790 (40.6)	86 (57.2)	<0.001
BBB^*∗∗*^, *n* (%)	50 (2.7)	35 (15.1)	<0.001	49 (2.7)	35 (12.3)	<0.001	57 (2.9)	26 (20.2)	<0.001	60 (3.1)	26 (17.1)	<0.001
3-vessel disease (%)	472 (25.3)	91 (39.6)	<0.001	455 (25.1)	107 (37.6)	<0.001	496 (25.2)	64 (50.3)	<0.001	508 (26.1)	70 (46.4)	<0.001
Left main stenosis, *n* (%)	114 (6.1)	20 (8.7)	0.072	110 (6.1)	24 (8.3)	<0.001	116 (5.9)	14 (10.9)	0.007	122 (6.3)	14 (9.1)	<0.001
Stent (%)	2432 (95.2)	193 (84.1)	<0.001	1723 (95.1)	246 (86.3)	<0.001	1866 (94.8)	140 (76.5)	<0.001	1910 (98.2)	120 (79.3)	<0.001
Postprocedural TIMI<3 (%)	83 (3.2)	43 (18.8)	<0.001	60 (3.3)	44 (15.3)	<0.001	65 (3.3)	37 (28.9)	<0.001	66 (3.4)	16 (10.8)	<0.001
CK, med (IQR)	1842 (9215, 3399)	2697 (1031, 5124)	<0.001	1820 (908, 3392)	2445 (1046, 4593)	<0.001	1864 (1007, 3387)	2456 (1051, 4033)	<0.001	1849 (925, 3471)	2189 (1004, 4355)	<0.001
Troponin I, med (IQR)	30.5 (9.8, 86.2)	41.3 (8.36, 11.89)	<0.001	30.6 (9.5, 86)	36.5 (8.4, 110.2)	<0.001	30.3 (9.5, 87.7)	47.4 (11.8, 111.6)	<0.001	30 (9.1, 86.1)	47 (12.5, 110.2)	<0.001
Haemoglobin g/L med (IQR)	141 (132, 152)	143 (130, 153)	0.050	142 (132, 153)	143 (129, 152)	<0.001	143 (132, 152)	136 (124, 149)	0.135	142 (132, 153)	135 (121, 147)	<0.001
LVEF med (IQR)	50 (44 55)	40 (30 50)	<0.001	50 (40, 55)	40 (35, 50)	<0.001	50 (40, 55)	30 (25, 40)	<0.001	50 (40, 55)	40 (25, 45)	<0.001
CrCl med (IQR)	92.4 (71.2, 114.9)	62.4 (45.2, 86.3)	<0.001	92.8 (71.8, 115.8)	77.8 (57.2, 97.4)	<0.001	91.11 (71.82, 111.37)	59.89 (45.32, 83.11)	<0.001	92.8 (71.8, 115.1)	63.3 (46.9, 90.2)	<0.001
RISK-PCI score, med (IQR)	3 (2, 5)	5.5 (4, 8)	<0.001	3 (2, 4.5)	5 (3, 7.5)	<0.001	3 (2, 5)	7.5 (5, 9.5)	<0.001	3 (2, 5)	7 (4.5, 9.5)	<0.001

Hours from symptom onset to first medical contact; ^*∗∗*^at admission; MACE = major adverse cardiovascular events; BBB = bundle branch block; MI = myocardial infarction; PCI = percutaneous coronary intervention; TIMI = Thrombolysis in Myocardial Infarction; LVEF = left ventricular ejection fraction; CrCl = creatinine clearance ml/min; MACE = major adverse cardiovascular events.

**Table 4:** Independent predictors for 1-year and 6-year MACE and mortality.

	HR	95% CI	*p* value
*1-year MACE*			
Killip class I and II at admission	1.60	(1.19–2.15)	0.002
Ejection fraction %	0.96	0.95–0.98	<0.001
RISK-PCI score	1.24	1.18–1.31	<0.001

*6-year MACE*			
Age, years	1.01	1.0–1.021	<0.001
Killip class II and III at admission	1.45	1.11–1.89	0.006
Ejection fraction %	0.96	0.95–0.98	<0.001
RISK-PCI score	1.22	1.16–1.28	<0.001

*1-year mortality*			
Age, years	1.03	1.01–1.04	<0.001
Killip class II and III at admission	2.08	1.41–3.09	<0.001
Anterior infarction	1.47	1.03–2.13	0.038
Ejection fraction %	0.92	0.90–0.94	<0.001
3-vessel disease	1.42	1.01–1.99	0.046
Leukocyte count	1.02	1.01–1.05	0.008
RISK-PCI score	1.21	1.13–1.29	<0.001

*6-year mortality*			
Age, years	1.03	1.02–1.05	<0.001
Killip class II and III at admission	1.91	1.35–2.70	<0.001
Anterior infarction	1.50	1.09–2.08	0.014
Ejection fraction %	0.92	0.91–0.94	<0.001
Leukocyte count	1.03	1.01–1.05	0.012
RISK-PCI score	1.23	1.15–1.31	<0.001

MACE = Major adverse cardiovascular events; HR = hazard ratio; CI = confidence interval.

## Data Availability

The data used to support the findings of this study are available from the corresponding author upon request.
